# Tunable Luminescent A-SiN_x_O_y_ Films with High Internal Quantum Efficiency and Fast Radiative Recombination Rates

**DOI:** 10.3390/ma11122494

**Published:** 2018-12-08

**Authors:** Pengzhan Zhang, Leng Zhang, Xuefeng Ge, Sake Wang

**Affiliations:** 1 College of Electronic and Information Engineering, Jinling Institute of Technology, Nanjing 211169, China; zhanglengxixi@163.com; 2 Collaborative Innovation Center of Advanced Microstructures, National Laboratory of Solid State Microstructures, Nanjing University, Nanjing 210093, China; 3 Center for Analysis and Testing, Nanjing Normal University, Nanjing 210023, China; gexuefeng@njnu.edu.cn; 4 College of Science, Jinling Institute of Technology, Nanjing 211169, China; IsaacWang@jit.edu.cn

**Keywords:** a-SiN_x_O_y_, N‒Si‒O defect states, photoluminescence internal quantum efficiency, radiative recombination rates, quasi-three-level systems

## Abstract

In this work, we systematically investigated the *N*_x_ bonding defects that induced high photoluminescence internal quantum efficiencies (PL IQEs) and very fast radiative recombination processes in amorphous silicon oxynitride (a-SiN_x_O_y_) systems. The luminescent N‒Si‒O bonding-related defect states were checked for the XPS, EPR, and temperature-dependent steady-state PL (TD-SSPL) properties. The PL IQEs were calculated from PL quantum yields through the principle of planar geometry optics, and then confirmed by the TD-SSPL properties. The radiative recombination rates [*k_r_*(R)] were determined by combining the PL IQE values and ns-PL lifetimes obtained from time-resolved PL measurements. Both the PL IQE, exceeding 72%, and the fast *k_r_*(R) (~10^8^ s^−1^) are proportional to the concentration of *N_x_* defects, which can be explained by N‒Si‒O bonding states related to the quasi-three-level model, suggesting the possible realization of stimulated light emission in a-SiN_x_O_y_ systems.

## 1. Introduction

As one of the traditional semiconductors, silicon (Si) is widely used in today’s microelectronic, photovoltaic, and optoelectronic technologies [1,2,3]. In Si-based monolithic optoelectronic integrated circuits, the most difficult work is to realize an efficient Si-based lighting source. However, silicon is not a suitable luminescent material, and its indirect band gap limits light emission efficiency. Therefore, Si-based luminescent materials (including Si alloys and nanostructured Si) have been actively investigated over the last two decades, with an interest in improving the PL external quantum efficiency (PL EQE, or called PL quantum yield, PL QY) and internal quantum efficiency (PL IQE), and understanding the radiative recombination mechanisms of the light emission [4,5,6,7,8,9,10,11,12,13,14,15,16,17,18,19,20,21,22].

Abundant previous works have reported on the improved light emission efficiency from colloidal Si QDs, and the results obtained from colloidal Si QDs were rather high (PL QY, 43–90%) [6,7,8]. However, only a few works have focused on the light emission efficiency in nanocrystal-Si embedded Si alloys. The PL EQE from thermal annealed nc-Si embedded Si nitride films, and PL IQE from nc-Si embedded Si dioxide films have been achieved at ~7% [10] and 59% ± 9% [11], respectively. On the other hand, although much attention has been paid to Si alloys [13,14,15,16,17], promising results are still lacking. Our group has found that O atoms’ impurity induced a significant enhancement of PL intensities in a-SiN_x_ films, and confirmed the new luminescent *N*_x_ defect centers [18,19,20]. Visible light emission devices with phosphorus-doped n-a-SiN_x_O_y_/p-Si heterojunction structure have also been realized [21]. Recently, PL EQE values of 8.38% have been achieved from tunable luminescent a-SiN_x_O_y_ films [22].

In this work, we systematically analyzed the PL internal quantum efficiencies and fast radiative recombination processes of a-SiN_x_O_y_ films. We checked the N‒Si‒O bonding defects using XPS, EPR, and temperature-dependent steady-state PL (TD-SSPL) measurements. The PL IQEs were calculated from the measured PL QY values using the principle of planar geometry optics, and then confirmed by the TD-SSPL properties. At the PL peak energy (*E*_PL_) of 2.55 eV, we achieved a high PL IQE of 72% in a-SiN_x_O_y_ systems. Combing with the obtained PL IQEs and ns-PL lifetimes, the fast radiative recombination rates (~10^8^ s^−1^) from a-SiN_x_O_y_ films have also been determined. Both the PL IQE values and the fast radiative recombination rates from a-SiN_x_O_y_ films for various stoichiometries are proportional to the related concentration of *N*_x_ defect states, which can be explained by a quasi-three-level model, suggesting the possible realization of stimulated light emission in a-SiN_x_O_y_ films.

## 2. Materials and Methods

### 2.1. Material Fabrication

A-SiN_x_O_y_ films with various thicknesses (10 nm–4 μm) and the controlled a-SiN_x_ were deposited on roughened quartz and p-Si substrates by using PECVD, by processes described in detail elsewhere [22]. N/Si ratios were effectively controlled through controlling the gas flow rate R (R = NH_3_/SiH_4_) by changing the ammonia flow. As the PL intensities increased rapidly when the film thickness increased, we chose ensemble samples with the same thickness of ~500 nm as the research objects in this article.

### 2.2. Characterization of A-SiN_x_O_y_ Thin Films

The chemical compositions and atomic scale defect states were confirmed by the XPS (Thermo ESCALAB 250, ThermoFisher Scientific, Waltham, MA, USA) and the EPR (Bruker EMXplus, X-band, Bruker, Billerica, MA, USA) measurements. The TD-SSPL and PL excited (PLE) properties were measured by a Fluorolo-3 system (HORIBA Jobin Yvon, Paris, France) in a computer-controlled Delta 9023 oven (State College, PA, USA) under various temperatures, using a 75W Xe lamp (*λ_exc_* = 250‒800 nm) and a He‒Cd laser (*λ_exc_* = 325 nm) as light sources. The optical band gaps (*E_opt_*) were obtained from transmittance measurements (Shimadzu UV-3600, Shimadzu Corp., Hadano, Kanagawa, Japan). The refractive indexes (*n*) were measured using a spectroscopic ellipsometer (Jobin Yvon UVISEL, HORIBA Jobin Yvon, Paris, France). Both the *E_opt_* and *n* of a-SiN_x_O_y_ samples are listed in Table 1. A FLS980 (Edinburgh Instrument Ltd., Edinburgh, UK) equipped with an EPL375 pulse diode laser (*λ_exc_* = 375 nm, pulse width ~53 ps, repetition rate ~20 MHz), and a TCSPC (resolution time ~100 ps), were used to record the time-resolved PL.

## 3. Results and Discussion

### 3.1. Identification of the N‒Si‒O Defect States

**Figure 1:**
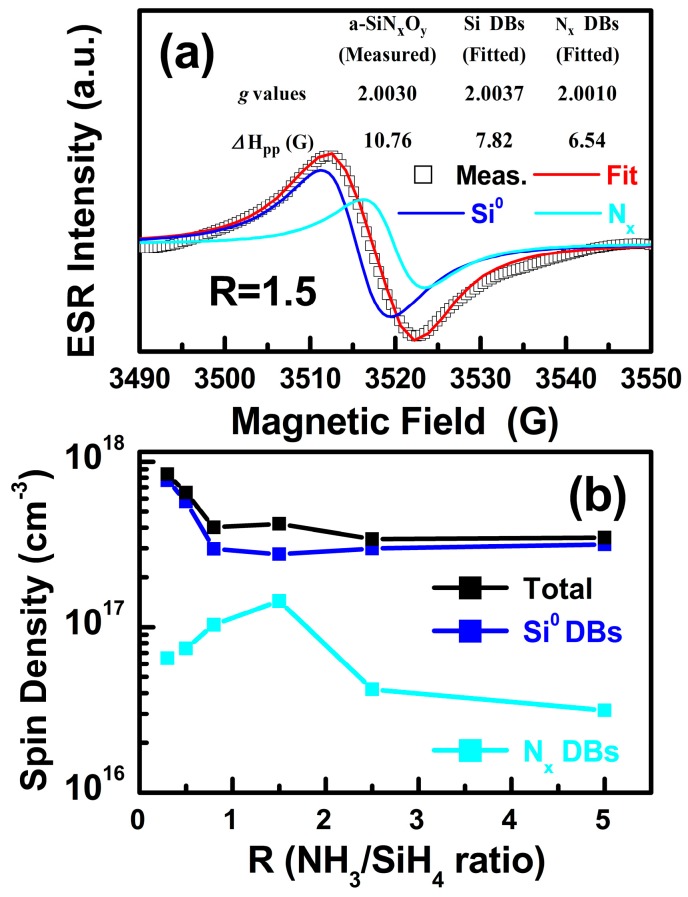
(**a**) The measured EPR spectra of a-SiN_x_O_y_ samples (R = 1.5) and the related simulations. (**b**) The total defects, Si DBs, and *N_x_* defects spin densities vs. R.

The existence of N‒Si‒O bonding configurations in a-SiN_x_O_y_ samples was confirmed by the XPS measurements [19,20,22]. The concentrations of N and O are 35–49% and 2.2–5.5%, respectively, for a-SiN_x_O_y_ films with various R. To further identify the related atom-scale defect states, we performed EPR measurements. Figure 1a displays the measured EPR absorption spectrum of the a-SiN_x_O_y_ films (R = 1.5), which has a value of *g* = 2.0030. We considered all possible typical trivalent Si dangling bonds (Si DBs) [23], and the nitroxide-like *N_x_* center [24], which is the herald of a true a-SiN_x_O_y_ phase [25]. Then we decomposed the measured EPR signals and obtained the related *g* values and Gauss line width (*∆*H*_pp_*), as shown in the Figure 1a inset. The densities of the coexisting Si DBs and *N_x_* defects of a-SiN_x_O_y_ films with various R can be calculated by doubly integrating all the measured and fitted first derivative EPR signals, which are plotted in Figure 1b.

### 3.2. PL Properties of the A-SiN_x_O_y_ Thin Films

**Figure 2:**
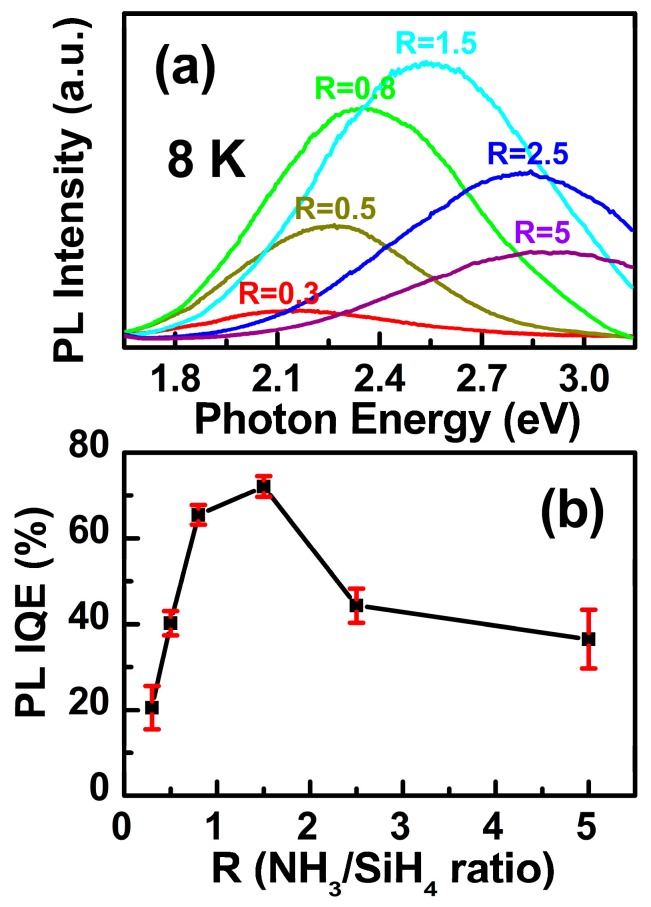
(**a**) The PL properties of a-SiN_x_O_y_ samples for various R at 8 K. (**b**) The related PL IQEs of a-SiN_x_O_y_ samples vs. R at RT.

For SSPL properties, with a rise in R, the PL peak positions exhibit a blue shift, and are tunable in the visible range (2.12–2.91 eV) under the excitation wavelength *λ_exc_* = 325 nm (He‒Cd laser), as shown in Figure 2a. Generally, the relationship between PL IQE (*ε*) and PL EQE (*η*) can be described as $\varepsilon =\eta /N^{*}$. Here $N_{}^{*}$ denotes the light extraction factor. We directly measured the absorption photon numbers and the emitted photon numbers in a calibrated integrating sphere, thus obtaining PL EQEs (PL QYs) exceeding 1.5% for tunable luminescent a-SiN_x_O_y_ films [22]. From the principles of planar geometry optics, we know that the emitted photons generated inside the samples are partially influenced by total internal reflection, and most of the generated photons are trapped inside the samples, since the refractive index of a-SiN_x_O_y_ samples (*n*) is much higher than the air index ($n_{air}$). Thus, the first part of light extraction factor $N_{1}^{*}$ can be calculated as [26,27]:

(1) $$N_{1}^{*}\approx \left[1-{\left(\frac{n-n_{air}}{n+n_{air}}\right)}^{2}\right]\times \frac{1}{2}\left(1-\sqrt{1-{\left(\frac{n_{air}}{n}\right)}^{2}}\right).$$

The remaining photons inside the sample should directly stroke or be internally reflected onto the rough substrate surface, which can also be scattered in all directions, and then emit out from the top surface [26,27], thus contributing the second and third parts of the light extraction factor $N_{2}^{*}$ and $N_{3}^{*}$, respectively. The rest of the contributions were too weak to separate out, and should be ignored. Therefore, the light extraction factor was defined as $N^{*}\approx \sum_{x=1}^{3}N_{x}^{*}$, and the calculated PL IQEs exceeding 20% should be obtained in the visible range, as shown in Figure 2b and Table 1. For various R, the variation tendency of *ε* was consistent with the variation tendency of *N*_x_ defect densities, indicating that luminescent *N*_x_ defects are responsible for the high PL internal quantum efficiencies in our a-SiN_x_O_y_ systems.

**Figure 3:**
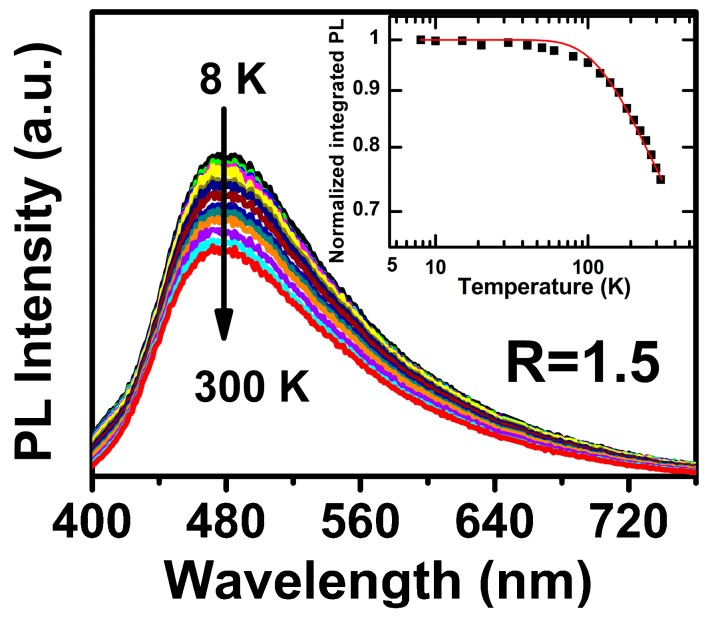
TD-SSPL spectra of a-SiN_x_O_y_ samples with R = 1.5. The inset exhibits normalized integrated PL intensities and the related simulations vs. measurement temperatures.

TD-SSPL measurements are always used to identify the PL mechanisms, and we check the related PL IQE values, as the radiative recombination makes a dominant contribution to the recombination processes at low temperatures, which means PL IQE is nearly equal to 100% [5,6,12]. Figure 3 shows the TD-SSPL properties for the R = 1.5 samples from 8 K to 300 K. One can see that the *E*_PL_ keeps nearly stable and is independent on the measurement temperatures. The PL profiles were observed no appreciable change under various measurement temperatures. Such phenomenon indicates that the carrier recombination through defect states, which is different from those through band tail levels. It will be discussed in detail later. As shown in Figure 3 insert, the integrated PL intensity [*I*_PL_(*T*)] keeps nearly stable at low measurement temperature range [T< 80 K, here we called *I*_PL_(*T*_0_)], and then decreased rapidly as the measurement temperature rises up from 80 K to 300 K, indicating the increasing domination of nonradiative recombination in this temperature range. We estimated the TD-PL IQE by using the thermal ionization model [10,13]:

(2) $$I_{PL}(T)=\frac{I_{PL}(T_{0})}{1+B\exp(-E_{a}/KT)},$$

where *B* is inversely proportional to the radiative recombination rates and *E_a_* denotes the activation barrier energy. From the thermal ionization model, *I*_PL_(T) was well fitted with *B* = 10 and *E_a_* = 57 meV, which was similar to the reported results [10]. The PL IQE for a-SiN_x_O_y_ films with R = 1.5 estimated from TD-SSPL measurements is 74.8%, which is consistent with the calculated values (*ε* ~72.1%) from directly measured PL QYs, and is much higher than those from nc-Si-embedded a-SiO_x_ samples [11].

### 3.3. Recombination Rates of A-SiN_x_O_y_ Thin Films

**Figure 4:**
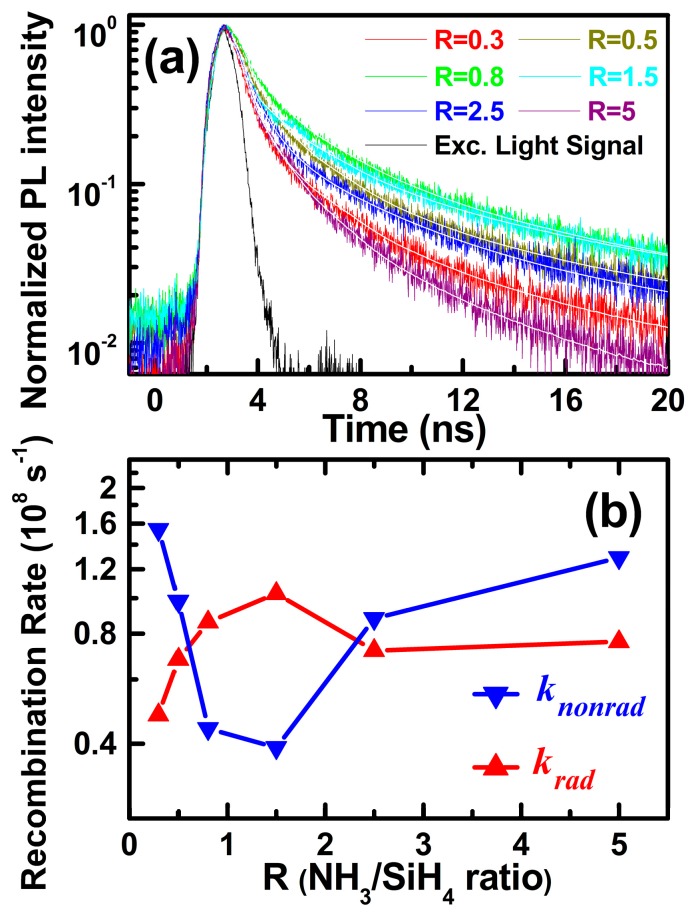
(**a**) ns-TRPL decay curves and the related simulations with different R values at RT. (**b**) The calculated *k*_r_(R) and *k*_nr_(R) of a-SiN_x_O_y_ films vs. R.

To further analyze the obtained high light emission efficiencies, we intensively investigated the ns-PL decay properties and the recombination rates [*k*(R)]. Figure 4a shows the ns-TRPL decay spectra of a-SiN_x_O_y_ samples at room temperature. We fitted the PL decay curves and obtained the ns-PL lifetimes (*τ_meas_*) by [28].

(3) $$I(t)={\sum_{i=1}^{n}A_{n}\exp(-t/\tau_{n})}_{}\;with\;\tau_{meas}=\frac{A_{1}\tau_{1}^{2}+A_{2}\tau_{2}^{2}+A_{3}\tau_{3}^{2}}{A_{1}\tau_{1}+A_{2}\tau_{2}+A_{3}\tau_{3}}.$$

The a-SiN_x_O_y_ samples for different R have an average value of about 6.23 ns at RT. Generally, *k*(R) can be expressed as $k\left(R\right)=k_{r}\left(R\right)+k_{nr}\left(R\right)$, where *k_r_*(R) and *k_nr_*(R) denote the radiative recombination rates and nonradiative recombination rates of a-SiN_x_O_y_ samples with various R, respectively. The radiative rates and nonradiative rates can be described as

(4) $$k_{r}\left(R\right)=\frac{1}{\tau_{r}\left(R\right)}=\frac{\varepsilon \left(R\right)}{\tau_{meas}\left(R\right)}\;with\;k_{nr}\left(R\right)=\frac{1}{\tau_{nr}\left(R\right)}=\frac{1-\varepsilon \left(R\right)}{\tau_{meas}\left(R\right)},$$

where *ε*(R) denotes the PL IQEs, and *τ_r_*(R) and *τ_nr_*(R) denote the radiative and nonradiative lifetimes, respectively. Combining the obtained *ε*(R) with the measured ns-PL lifetimes, *k_r_*(R) and *k_nr_*(R) were calculated from Equation (4), as shown in Figure 4b. The *k_r_*(R) have an average value of about 0.8 × 10^8^ s^−1^ under RT, which can be compared with that of direct band gap materials (such as CdSe NCs, *k_r_* ~10^8^ s^−1^ [29]). We found that the variation tendency of the radiative recombination rates was also consistent with the variation tendency of *N*_x_ defect densities, thus contributing to our understanding of the domination of N‒Si‒O bonding defect states in fast radiative recombination processes.

### 3.4. A-SiN_x_O_y_ Quasi-Three-Level Systems

We further intensively studied the *N*_x_ defect features of luminescent a-SiN_x_O_y_ films by analyzing the variation tendency of PL peak positions (*E*_PL_) for change of excitation photon energies (*E*_exc_), comparing with those of the controlled a-SiN_x_ films.

**Figure 5:**
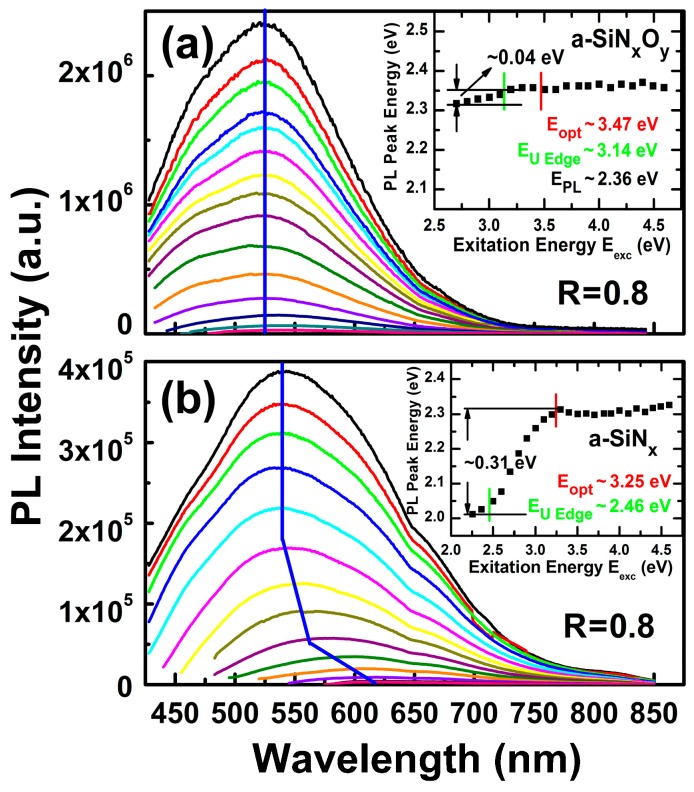
PL spectra of (**a**) a-SiN_x_O_y_; (**b**) a-SiN_x_ samples with R = 0.8 under different *E*_exc_. Insets exhibit the PL peak positions vs. *E*_exc_.

Different PL spectra of a-SiN_x_O_y_ films with R = 0.8 under different *E*_exc_ are given in Figure 5a. We found that the *E*_PL_ of a-SiN_x_O_y_ films hardly varied (~0.04 eV) for change of excitation photon energies, whether *E*_exc_ > *E*_opt_ or *E*_exc_ < *E*_opt_. We also see that band shape profiles are not changed for different *E*_exc_; this is a typical feature of luminescence related to defect states. For the PL spectra of the controlled a-SiN_x_ films in Figure 5b, in the case of *E*_exc_ < *E*_opt_, the *E*_PL_ of a-SiN_x_ films has a blue shift (~0.31 eV); when *E*_exc_ rises, in the case of *E*_exc_ > *E*_opt_, *E*_PL_ stays nearly constant, exerting the typical PL characteristics of the radiative recombination from band tail states carrier transition.

Then we calculated the Stokes shifts from conduction band tails (*E*_U *Edge*_) to PL peak positions ($\Delta E_{stokes}=E_{U}{{}_{}}_{Edge}-E_{PL}$). It is generally known that PLE spectra represent the state density distribution of luminescence excitation states (conduction band *E_C_*); thus we can analyze the excitation processes and PL excitation mechanisms from PLE spectra. Excitation states of amorphous semiconductors involve band tail states of *E_C_* extended in the band gap; this represented the *E*_U *Edge*_ of the *E_C_* tail, which is defined as the boundary threshold value energy of PLE. *E_C_* tail width *E*_U_ can also be estimated by subtracting the *E*_U_ *_Edge_* from the *E_C_* (*E_opt_*) of samples, i.e., $E_{U}=E_{opt}-E_{U_{}Edge}$. The *E*_PL_ continues to have a blue shift with a sustained rise in R for tunable luminescent a-SiN_x_O_y_ films in the visible range. Firstly, we noticed that a blue shift occurred to the *E*_PL_ and *E_opt_* was broadened with the increase of R, while *E*_U *Edge*_ would shrink in the direction of *E_C_* (*E*_opt_) energy level with the increase in N content, and the position of N‒Si‒O defect states energy level in a-SiN_x_O_y_ films was fixed under *E*_U *Edge*_. For a-SiN_x_O_y_ films with different R, Stokes shift has no variation with changes in *E_opt_*, and converges to a stable value ($\Delta E_{stokes}$~0.8 eV), as shown in Table 1. On the other hand, Robertson et al. reported that the Si‒N bond would form a bonding state (*σ*) and an anti-bonding state (σ^*^), and *E_opt_* was calculated from the differences between these two energy levels in the a-SiN_x_ band gap [30]. For a-SiN_x_ with x < 1.2, the position of *E_C_* bottom would basically not change with the content of N, while the movement of the valence band (*E_V_*) in the top Si‒N bond caused a change of *E*_opt_. With the increase in N content (x > 1.25), the *E_C_* bottom of a-SiN_x_ films was gradually replaced by Si‒N anti-bonding states with a higher energy position, and the *E_V_* top was gradually replaced by a lone-pairs state of N 2p; at this point, the increase in *E*_opt_ was mainly decided by the movement of the *E_C_* bottom to a higher energy level. For our a-SiN_x_O_y_ films, from the XPS measurements, the x of N have a range of about 0.54–0.96 with various R. Therefore, combining the factors of the aforementioned aspects, we assumed that the *E*_PL_ of N‒Si‒O-related defect states would shift through control of N content (varying with changes in *E*_opt_), which is caused by the movement of the *E_V_* top in the band gap in a-SiN_x_O_y_ films.

**Figure 6:**
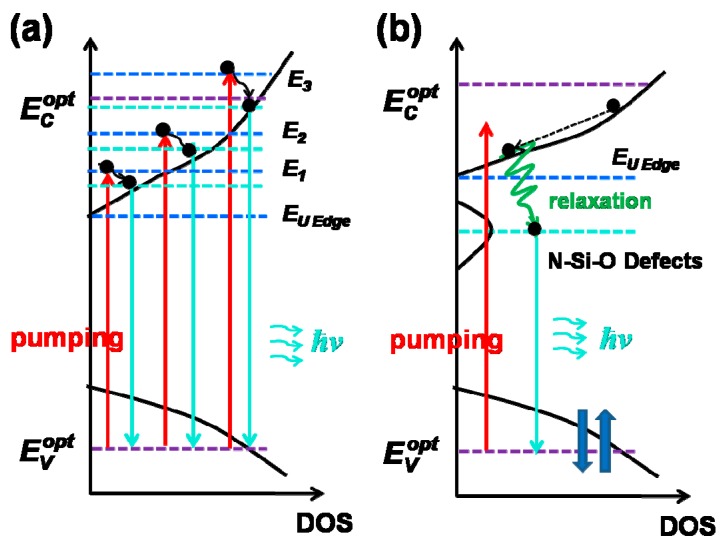
The schematic diagrams of luminescence model and transition of electrons in the band gap: (**a**) a-SiN_x_; (**b**) a-SiN_x_O_y_.

Therefore, based on the research into luminescent *N_x_* defects and related ns-TRPL decay above, we put forward a quasi-three-level systems model to explain the obtained high PL IQEs (*ε* ~72%) and fast radiative recombination rates (*k_r_* ~10^8^ s^−1^) in a-SiN_x_O_y_ systems, which is distinctly different from those of band tail related a-SiN_x_ systems [13]. The typical PL mechanisms of band tail state carrier transitions are shown in Figure 6a. When *E*_exc_ is less than *E*_opt_ (*E*_exc_ < *E*_opt_), excitation state carriers (*E*_1_) relax to a deeper energy level, and achieve recombination luminescence among bands after thermalization. With the rise in *E*_exc_, excitation state carriers will occupy a higher energy level of conduction band tail (from *E*_1_ to *E*_2_), so that *E*_PL_ should move to the location of the high energy level by degrees, i.e., *E*_PL_ blue shift. In case *E*_exc_ > *E*_opt_, *E*_PL_ tends to stay stable and is independent of *E*_exc_. However, for a-SiN_x_O_y_ systems, as shown in Figure 6b, the radiative recombination processes should be divided into two steps: firstly, excitation state electrons relax to the band tail of conduction band in the process of nonradiative recombination, and are caught by N‒Si‒O relevant defect center after thermalization; then electrons transit from N‒Si‒O defect states to the valence band to conduct radiative recombination transition, resulting in highly efficient light emission and fast radiative recombination rates.

## 4. Conclusions

In conclusion, the PL internal quantum efficiencies and radiative recombination mechanisms have been investigated in tunable luminescent a-SiN_x_O_y_ systems. PL IQEs of 72% have been achieved, which is much higher than those of nanocrystal Si-embedded Si oxide films. Fast radiative recombination rates (~10^8^ s^−1^) have also been achieved. We discussed and put forward the PL mechanisms of luminescent N‒Si‒O defect-related quasi-three-level systems, which suggested the possibility of stimulated light emission in a-SiN_x_O_y_ films.

## Figures and Tables

**Table 1:** The optical parameters, *E*_PL_ (*E_exc_* > *E_opt_*), PL QEs, and ns-PL lifetimes of a-SiN_x_O_y_ films.

R	*E_opt_* (eV)	*E*_U Edge_ (eV)	*E*_PL_ (eV)	Δ*E*_stokes_ (eV)	*n*	N* (%)	*η* (%)	*ε* (%)	*τ_meas_*(ns)
0.3	2.93	2.86	2.12	0.74	2.265	7.64	1.57	20.5	5.18
0.5	3.15	3.02	2.23	0.79	1.966	10.77	4.33	40.2	6.07
0.8	3.47	3.14	2.36	0.78	1.904	11.63	7.76	65.5	7.12
1.5	3.98	3.38	2.55	0.83	1.889	11.85	8.38	72.1	7.79
2.5	4.50	3.66	2.81	0.85	1.837	12.67	5.61	44.3	6.31
5	4.62	3.75	2.91	0.84	1.803	13.25	4.84	36.5	4.91
